# Nutrient Removal and Uptake by Native Planktonic and Biofilm Bacterial Communities in an Anaerobic Aquifer

**DOI:** 10.3389/fmicb.2020.01765

**Published:** 2020-07-29

**Authors:** John T. Lisle

**Affiliations:** St. Petersburg Coastal and Marine Science Center, United States Geological Survey, St. Petersburg, FL, United States

**Keywords:** nutrient uptake, biofilms, phosphorus, nitrogen, carbon, groundwater, managed aquifer recharge, aquifer storage and recovery

## Abstract

Managed aquifer recharge (MAR) offers a collection of water storage and storage options that have been used by resource managers to mitigate the reduced availability of fresh water. One of these technologies is aquifer storage and recovery (ASR), where surface water is treated then recharged into a storage zone within an existing aquifer for later recovery and discharge into a body of water. During the storage phase of ASR, nutrient concentrations in the recharge water have been shown to decrease due, presumably via the uptake by the native aquifer microbial community. In this study, the native microbial community in an anaerobic carbonate aquifer zone targeted for ASR storage was segregated into planktonic and biofilm communities then challenged with NO_3_-N, PO_4_-P, and acetate as dissolved organic carbon (DOC) to determine their respective removal and uptake rates. The planktonic community removed NO_3_-N at a rate of 0.059 mg L^–1^d^–1^, PO_4_-P at 5.73 × 10^–8^–1.03 × 10^–7^ mg L^–1^d^–1^ and DOC at 0.015–0.244 mg L^–1^d^–1^. The biofilm community was significantly more proficient, removing NO_3_-N at 0.116 mg L^–1^d^–1^ (1.6–9.0 μg m^–2^d^–1^), PO_4_-P at 4.20–5.91 × 10^–5^ mg L^–1^d^–1^ (2.47–9.88 ng m^–2^d^–1^) and DOC at 0.301–0.696 mg L^–1^d^–1^ (29.0–71.0 μg m^–2^d^–1^). Additionally, the PO_4_-P sorption rate onto the carbonate aquifer matrix ranged from 1.64 × 10^–7^ to 9.25 × 10^–7^ mg PO_4_-P m^–2^ day^–1^. These rates were applied to field data collected at an ASR facility in central Florida and from the same aquifer storage zone from which the biofilm communities were grown. With only 10% of the available surface area within the storage zone being colonized by biofilms, typical concentrations of NO_3_-N, PO4-P, and DOC in the recharged filtered surface waters would be reduced to below detection limits, and by 81.4 and 91.1%, respectively, during a 150 days storage period.

## Introduction

The availability and quality of freshwater is becoming a global issue as sources are impacted by not only natural variability in precipitation but also the expansion of human habitation into wetlands and increases in agricultural, domestic and industrial demands (Koutroulis et al., 2019). One of the options available to water resource managers to recover and store excess freshwater until it’s needed is managed aquifer recharge (MAR) (Bekele et al., 2018). MAR is a collective term for technologies that inject a variety of treated surface and process waters into aquifer zones for later recovery (Bekele et al., 2018). One of these technologies is aquifer storage and recovery (ASR) (Pyne, 2005; Bekele et al., 2018). As part of the ASR optimization process a series of cycle tests are performed where treated source water is recharged into the aquifer storage zone, allowed to stay in the storage zone for a predetermined length of time and then recovered and discharged at the surface into a body of water. During the storage phase of the cycle tests its common for concentrations of constituents in the recharge water (e.g., bacteria, metals, nutrients, etc.) to be significantly reduced in the recovered water (Mirecki, 2004; Patterson et al., 2010; Mirecki et al., 2013; Vanderzalm et al., 2013, 2018; Page et al., 2017). For example, the concentrations of NO_x_-N, PO_4_-P and TOC in recharged surface water were reduced during the storage phase in an anaerobic aquifer by up to 100.0, 81.4, and 91.1%, respectively (Mirecki, 2013) and by 100.0, 49.4, and 54.1% in recharged stormwater stored in an anoxic aquifer (Vanderzalm et al., 2018). These reduction rates are derived from net removal data of the respective constituents after storage, regardless of the storage time interval. Additionally, biogeochemical processes are assumed to be the dominant, with geochemical reactions being a minor, contributor to most of the removal rates during the storage phase.

The biogeochemical or microbial processes responsible for the reduction in constituents in the recharge water are initially being performed by bacteria native to the aquifer storage zone with the diversity and possibly physiological function being altered after repeated recharge-storage-recovery cycles (Ginige et al., 2013). There is a consensus the vast majority of these processes are associated with the biofilm communities, in contrast to the planktonic communities, in the storage zones. This physiological dominance is due to biofilms having been shown to always contain relatively greater numbers of bacterial cells than in the planktonic phase of the same system (Whitman et al., 1998). Biofilm associated cells in groundwater ecosystems (1.4 × 10^30^) have been estimated to exceed that of the planktonic cells (5.0 × 10^27^), on a global basis, by several orders of magnitude (McMahon and Parnell, 2014; Flemming and Wuertz, 2019).

In this study, the native microbial community in an anaerobic and reduced zone of the Upper Floridan Aquifer (UFA) (Miller, 1997; Morrissey et al., 2010), that has been targeted as an ASR storage zone, was segregated into planktonic and biofilm communities. These communities were then separately challenged with concentrations of NO_3_-N, PO_4_-P and dissolved organic carbon (DOC) commonly found in ASR source surface water in south-central Florida. Removal rates for NO_3_-N and uptake rates for PO_4_-P and DOC were derived from data collected under native groundwater conditions and represent baseline removal and uptake rates for the native microbial planktonic and biofilm communities living in this zone of the UFA.

## Materials and Methods

### Sample Site Location and Hydrogeology

The artesian groundwater source well (27° 09′ 17.3′′; 80° 52′ 27.4′′ W) is located within the Kissimmee River ASR (KRASR) facility located near the confluence of the Kissimmee River and Lake Okeechobee (Mirecki, 2013; Mirecki et al., 2013). This well is 0.254 m diameter steel cased to 174.3 mbls with a single screened collection zone between 174.3 and 268.2 mbls. The collection zone is within the artesian Upper Floridan Aquifer (UFA) that is characterized as a thick sequence of interlayered marine calcareous and dolomitic limestones of Eocene and Oligocene age, overlain by a confining unit consisting of approximately 122 m of Hawthorn Group interlayered clays, silts, and fine sands (Scott, 1988). The lower confining layer consists of 122–152 m of dolomitic limestone, dolomite, and dolostone (Golder Associates, 2007; Reese and Richardson, 2007; Waldron and Horvath, 2010). These confining units isolate this zone of the UFA from other groundwater sources positioned above or below (Miller, 1997). Additionally, the collection zone is not impacted by meteoric or surface water as the isotopic age of the groundwater in this region of UFA has been estimated at approximately 2.5 × 10^4^ years since it was first recharged into the subsurface (Plummer and Sprinkle, 2001). The permeability within this zone of the UFA is not uniform as 92% of the total flow occurs in two depth intervals at 166.4–185.6 mbls (80%) and 268.2–283.5 mbls (12%). The storage zone is positioned between these two intervals at 166.0–261.0 mbls. The recharged water within this storage zone is nominally-to-unaffected (i.e., not diluted) during the storage phase based on chloride-based conservative mixing modeling (Mirecki et al., 2013). An aquifer performance test on the collective aquifer zone accessed during this study produced a transmissivity of 3,416 m^2^ d^–1^ (Reese and Richardson, 2007).

### Sample Container Preparation

All reactors, glass and plasticware, fittings and closures used in these experiments were first washed with laboratory detergent, rinsed in tap water, rinsed in reagent grade water, soaked overnight in a 10% (v/v) HCl solution, rinsed three times with reagent grade water and allowed to air dry. Once dry, all glassware and closures were sterilized by autoclaving then transferred to an anaerobic chamber with a N_2_/CO_2_/H_2_ (85%:10%:5%) atmosphere and allowed to degas for a minimum of 2 days before use. Prior to removal from the anaerobic chamber for transport to the research site, all fittings and closures were secured on the respective pieces of glassware and polypropylene containers then placed in gas tight containers for transport.

### Groundwater Chemistry

The general geochemistry and nutrient data (mean ± SD) for the zone of the UFA accessed during this study were taken from four wells located on the KRASR facility property previously reported (Mirecki, 2013) (Table 1). Additionally, the NO_3_-N and dissolved organic carbon (DOC) concentrations used in the calculations of the removal rates during storage were taken from the KRASR pilot study cycle test data (i.e., an ASR cycle is the recharge, storage and recovery of treated surface water into and from the aquifer zone) (Mirecki, 2013). The PO_4_-P concentration data were extracted from a figure in the KRASR pilot study report showing the trends in phosphorus concentrations during the recharge and recovery phases of a cycle test using WebPlotDigitizer^1^.

**TABLE 1:** Upper Floridan Aquifer geochemical data.

**Parameter**	**Units**	**Mean (±SD)**
Temperature	°C	25.56 ± 0.27
pH		7.89 ± 0.21
ORP	mV	−258.4 ± 30.75
Specific conductance	μS cm^–1^	1269.8 ± 156.32
Turbidity	NTU	0.45 ± 0.36
Color	PCU	5.85 ± 1.2
Total dissolved solids	mg L^–1^	727.8 ± 110
Total alkalinity (as CaCO_3_)	mg L^–1^	85.2 ± 4.58
Aluminum	μg L^–1^	5.65 ± 7.99
Barium	μg L^–1^	29.02 ± 3.16
Boron	μg L^–1^	82 ± 18.38
Bromide	mg L^–1^	660 ± 138.2
Calcium	mg L^–1^	46.42 ± 4.11
Chloride	mg L^–1^	232.6 ± 50.96
Copper	mg L^–1^	1.38 ± 0.66
Fluoride	mg L^–1^	0.53 ± 0.04
Iron	μg L^–1^	90.17 ± 77.92
Magnesium	mg L^–1^	36.52 ± 2.72
Manganese	μg L^–1^	4.45 ± 1.93
Potassium	mg L^–1^	7.3 ± 1.52
Silica	mg L^–1^	8.2 ± 5.11
Sodium	mg L^–1^	137.14 ± 37.33
Sulfate	mg L^–1^	184.6 ± 12.66
Sulfide	mg L^–1^	1.07 ± 0.22
Zinc	μg L^–1^	9.72 ± 11.42
NO_2_-N	mg L^–1^	< 0.01^a^
NO_3_-N	mg L^–1^	< 0.03^a^
NH_3_-N	mg L^–1^	0.22
Total PO_4_-P	mg L^–1^	0.03
Ortho PO_4_-P	mg L^–1^	< 0.01^a^
Total organic carbon	mg L^–1^	1.7
Dissolved organic carbon	mg L^–1^	1.40 ± 0.28

*^*a*^Denotes the analytical detection limit.*

### Groundwater Sample Collection

Prior to sampling, the groundwater well was allowed to flush through a 10.2 cm diameter valve to waste until a minimum of three well casing volumes had been removed. The large volume valve was closed and a 2.0 cm diameter, stainless steel valve with a tubing fitting was opened at a laminar flow rate and allowed to flush to waste for several minutes before attaching a sterilized 10.0 L stainless steel pressure vessel (MilliporeSigma, Burlington, MA, United States), fitted with valves and hose connectors on the inflow and outflow ports. Groundwater was allowed to flow through the pressure vessel’s inflow and outflow ports to waste for a minimum of four volumes before sealing the groundwater sample from the atmosphere by turning both outflow and inflow valves off while ensuring there was no head space in the vessel. Collecting sub-samples of the collected groundwater was accomplished by pressurizing the vessel with either Ar or N_2_ gas, depending on the experimental design, while having all bench top microcosms under constant gas flow of the respective gases when dispensing sub-samples.

### Core Material for Biofilm Growth Substrate

Core material from the same well at the depth of the groundwater collection zone was acquired from the core archives maintained at the Florida Geologic Survey^2^. For the nitrogen species biofilm uptake experiments, the core material was cut into irregular shaped coupons with two smooth surfaces and consistent thickness that would fit into a biofilm microcosm as described below. All core coupons for the phosphorus and carbon biofilm uptake experiments were cut in dimensions of 1.21 cm (width) × 0.64 cm (thickness) × 2.54 cm (length). These coupons were sterilized and processed as described above. All coupons were loaded into the respective biofilm growth reactors, as described below, while still in the anaerobic chamber and prior to removal for transport to the research site. All coupons were sterilized by autoclaving (121°C, 15 psi, 15 min) three times, then placed in an anaerobic chamber with a N_2_/CO_2_/H_2_ (85%:10%:5%) atmosphere and allowed to degas for a minimum of 2 days.

Currently, there are no data on surface areas within the pore or channel networks of this or any other zone of the Floridan Aquifer System. However, the surface area within the core coupon upon which the biofilm would grow were estimated based upon a range of surface area-to-mass ratios for carbonate rock very similar to that in the UFA (Lai et al., 2015) and known specific densities for carbonate rock from the UFA in south Florida (Sunderland et al., 2011). The surface area-to-mass ratios ranged from 0.8–4.3 m^2^ g^–1^, and the specific gravity values were bimodal ranging from 2.70–2.79 and 2.81–2.83 g cm^–3^. Using the lower and upper limit specific gravity values to estimate the range of surface areas for biofilm growth within the core coupons, the core segments used in the NO_3_-N microcosm ranged from 12.85–72.37 m^2^ and 4.25–23.92 m^2^ for the coupons in the PO_4_-P and carbon microcosms.

### Biofilm Growth Reactors

The biofilm growth reactors for the nitrogen species uptake experiments were sterile borosilicate glass chromatography columns (2.5 cm × 30.0 cm). The core coupons were placed into the reactors in irregular orientations before being sealed on both ends with caps fitted with PTFE values with push-tube fittings. The reactors for the phosphorus and carbon uptake experiments were sterile 2.5 cm × 30.5 cm PTFE pipes. The core coupons were placed into these reactors end-to-end and in the same orientation before being sealed with PTFE values with push-tube fittings.

### Biofilm Growth System

**FIGURE 1:**
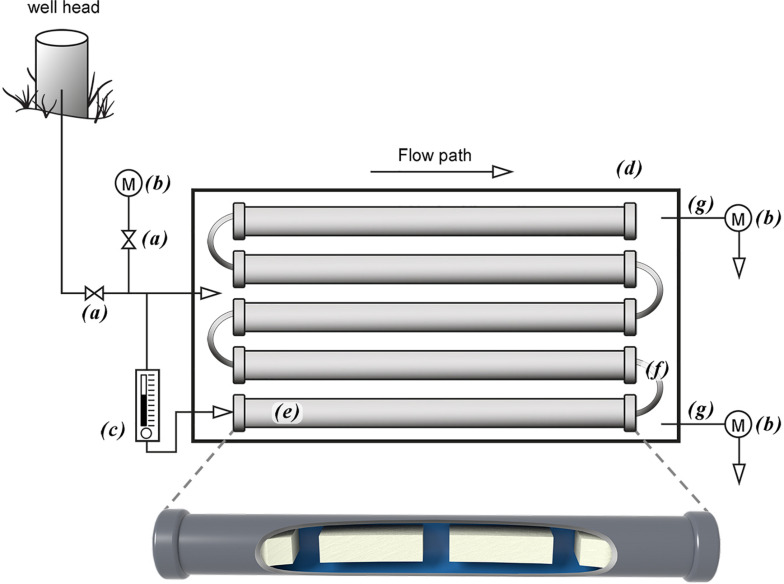
Above ground mesocosm with biofilm growth reactors. Groundwater from the well head is directly transferred to the mesocosm via PTFE tubing into a high flow rate control valve **(a)** that diverts groundwater into a flow cell containing a water quality measurement meter **(b)** and a low flow control valve **(c)**. Groundwater passing through the high flow control valve fills the outside chamber **(d)** while the low flow rate groundwater flows through a series of biofilm growth reactors that contain aquifer core coupons on which the biofilms grow **(e)** and connected by PTFE tubing **(f)**. Both flow systems discharge through PTFE tubing **(g)** into flow cells containing water quality measurement meters **(b)**.

A two chamber system was designed to allow groundwater to flow over the biofilm growth reactors that contain the core coupons, at close to *in situ* rates, while insulating the coupons from surface temperatures and exposure to air by groundwater flowing outside the reactors at high rates (Figure 1). High volume and flow rates though an insulated 340 L HDPE container (EW0632288; Cole-Parmer, Vernon Hills, IL, United States) were maintained through a black PTFE (1.27 cm OD) tubing connected to a stainless steel valved fitting on the well head and the other end slipped into a water tight fitting located at the bottom of the outside wall on one end of the container. Groundwater discharged from the container through a 5.1 cm diameter opening located at the top container in the wall opposite the inflow tubing.

Low groundwater volume and flow rates for biofilm growth were established via a 3-way valve that had been inserted into the high flow volume PTFE tube prior to entering the 340 L container. A low flow control valve (MR3000; Brooks Instrument, Hatfield, PA, United States) was connected to the 3-way valve via black PFTE tubing (0.47 cm OD) with the tubing from the out flow side of the flow control valve traversing the wall of the container through a water tight fitting and into one end a biofilm growth reactor via the push-tube fitting. Multiple biofilm growth chambers were connected in series using 5.0 cm pieces of the same tubing. A longer piece of the tubing was connected to the last biofilm growth chamber and though a water tight fitting located next to the groundwater discharge opening for the high volume flow. All valves on the biofilm growth reactors were then opened to initiate groundwater flow across the core coupons. The groundwater flow rate through the larger container was set at approximately 10.0 L min^–1^, while the flow rate through the biofilm growth reactors was 150.0 mL min^–1^. All reactors were left in place for approximately 10 months before removing for use in the biofilm microbial community uptake experiments described below.

### Biofilm Coupon Collection

The valves on both ends of the biofilm growth reactors recovered for an experiment were closed, disconnected from the tubing and immediately transferred to and submerged in a container of groundwater collected at the well head. The remaining growth reactors were left in series and maintained groundwater flow by reconnecting the last biofilm growth reactor in the series to the outflow tubing.

Once in the laboratory the biofilm growth reactors were removed from the groundwater container, the valves on either end opened, then connected to a gas source to maintain an anaerobic atmosphere around the biofilms. The groundwater in the reactor was gently drained while under constant Ar-gas flow for the nitrogen species uptake experiments and N_2_-gas for the phosphorus and carbon uptake experiments. Biofilm coupons were then aseptically retrieved with sterile forceps, gently dipped in filter sterilized groundwater to remove non-attached cells, then transferred to each of the biofilm benchtop microcosms. All containers and benchtop areas used in these procedures were aggressively flushed with Ar-gas flow for the nitrogen species removal experiments and N_2_-gas for the phosphorus and carbon uptake experiments.

### Nitrogen Removal by Planktonic and Biofilm Microbial Communities

A benchtop microcosm (500 mL borosilicate glass bottles), with gray bromobutyl rubber septa plugs (Chemglass Life Sciences), was flushed with Ar-gas prior to transferring groundwater from the pressurized sample vessel. Once adequately flushed, 450 mL of groundwater was transferred to the microcosm, then dosed with a standardized stock solution of KNO_3_ (12.50 mM; 1.26 g/L) to provide a final concentration of 25.0 μM (0.350 mg/L) NO_3_-N. A 50 mL sub-sample was immediately collected for the time zero sample before sealing and gently mixing the microcosm and incubating up-side-down at 25–27°C in the dark.

At each time point, approximately 50.0 mL of Ar-gas was injected into the headspace of the microcosm using a gas tight syringe, then approximately 40.0 mL of groundwater was removed using a 19G needle attached to a 60 mL syringe. Approximately two 20 mL volumes were filtered through a 0.22 μm pore size syringe filter into separate 35 mL HDPE bottles and immediately frozen at −80°C, then stored at −20°C until analysis.

All time point samples were analyzed for NO_2_-N, NO_3_-N and NH_4_-N using a Seal Analytical Auto Analyzer 3 employing the protocols of Gordon et al. (2000). Minor modifications of the ammonium technique were required to extend the dynamic range to 30 μM for anoxic and other high ammonium waters by reducing the respective flow rates for the nitroprusside (50.0 μL min^–1^), hypochlorite (50.0 μL min^–1^), phenolate (50.0 μL min^–1^), citrate (320.0 μL min^–1^), sample (600.0 μL min^–1^), air bubble (160.0 μL min^–1^), and waste draw (1200.0 μL min^–1^).

The benchtop microcosms for nitrogen species uptake by biofilm microbial communities were set up as described for the planktonic communities, with the exception that the groundwater was filter sterilized (0.22 μm pore size filtration) under Ar-gas flow before adding to each of the microcosms. Once each microcosm had been dosed with the nitrogen species stock solution as described for the planktonic microbial community biofilm coupons were aseptically removed under Ar-gas flow and transferred to the microcosms. Negative controls for the biofilm microbial community uptake microcosms were a set of biofilm coupons that had been immersed in formalin for 15 min before transferring to a 50 mL tube containing filter sterilized and dosed groundwater from the NO_3_ or NH_4_ benchtop microcosms. These samples were incubated with the benchtop microcosms and processed with the samples collected at the last time point. Sample collection and processing were the same as described for the planktonic microbial community microcosms.

Following the completion of the biofilm uptake experiments, the coupons were air dried before calculating the coupon surface area within each benchtop microcosm. Coupon surface areas were estimated by first tracing the outlines of the flat surfaces and edges of each coupon onto paper and cutting those outlined areas out as individual pieces. The weight of a 2.5 cm^2^ piece of the same paper was weighed to provide a surface area-to-weight conversion factor. This conversion factor was then used to convert the total weights of the coupon cutouts into surface areas.

### Phosphorus Uptake by Planktonic and Biofilm Microbial Communities

The benchtop microcosms (250 mL polycarbonate screw cap flask) were kept under constant N_2_-gas flow and filled with 110 mL of groundwater from the pressure vessel as previously described. To this volume the following were added (final concentration): cold potassium phosphate (1.32 nM; 0.125 μg L^–1^ as PO_4_; 0.44 nM; 0.042 μg L^–1^ as P), ^32^PO_4_ [0.132 nM; 12.50 ng L^–1^ PO_4_; 0.044 nM; 4.20 ng L^–1^ as P; 286.6 Ci mg^–1^ (ARC Inc., St. Louis, MO, United States)] for an approximate scintillation count of 2 × 10^6^ CPM 20 mL^–1^ sample and sodium acetate (0.831 mM, 68.17 mg L^–1^; 0.333 mM, 4.00 mg L^–1^ as C). Acetate was added based on preliminary experiments that had shown no measurable uptake of ^32^P after up to 6.0 h incubation at 25–27°C, without the addition of acetate. Acetate was chosen as this carbon source is most commonly found in anaerobic and reduced geochemical groundwater conditions similar to those in this zone of the UFA.

The suspension was gently mixed, and 10.0 mL immediately removed and transferred to a 15 mL polypropylene tube containing 500.0 μL of formalin (i.e., killed sample). The remaining volume in the microcosm was under N_2_-gas flow for the entirety of the experiment. At each time point 20.0 mL were removed from the microcosm, then 10.0 mL transferred to two 15 mL tubes as replicates. The entire volume of each replicate was filtered through a vacuum filtration system which captured the microbial biomass on a membrane filter (mixed cellulose ester, 25 mm, 0.22 μm pore size) (MilliporeSigma, Burlington, MA, United States) and the filtrate into a separate 15 mL tube.

After removing the filtrate collection tubes from the filtration system, the filters were rinsed three times with filter sterilized source groundwater, transferred to scintillation vials, allowed to air dry, then 5.0 mL of Ultima Gold (PerkinElmer, Waltham, MA, United States) scintillation cocktail added to each. A 1.0 mL sub-sample of each replicate’s filtrate was transferred to scintillation vials and 5.0 mL of the same scintillation cocktail was added to each. All samples were allowed to set at room temperature in the dark for 6–8 h to stabilize, then counted on a scintillation counter.

Groundwater (120.0 mL) of groundwater from the pressure vessel was filter sterilized (0.22 μm pore size) into a sterile and degassed flask under constant N_2_-gas flow, then dosed with cold potassium phosphate, sodium acetate and ^32^PO_4_ to final concentrations and activities described for the planktonic microbial community experiments. A set of 12 microcosms (sterile and degassed 50 mL polypropylene tubes) each received 10.0 mL of the filter sterilized and dosed groundwater while under constant N_2_-gas flow.

Biofilm growth reactors with the smaller, regularly cut coupons were recovered, transported and processed for delivery biofilm coupons as described for the nitrogen species uptake experiments. Each of 10 microcosms received a single biofilm coupon 2.5 cm in length, with one microcosm being immediately processed as described below for the time zero time point sample. The remaining microcosms were incubated upright at 25–27°C and in the dark.

Two control microcosms were set up for the ^32^PO_4_ uptake experiments: dosed filtered sterilized groundwater with no biofilm coupon and dosed filter sterilized groundwater into which a biofilm core coupon was transferred that had been inactivated (i.e., killed sample) by immersion in 10.0 mL of filter sterilized groundwater supplemented with 200 μL for 15 min before transfer to the microcosm. The two control experiments were incubated as described for the other tubes and collected and processed with the last time point samples.

For each time point, including time zero, one microcosm was recovered and processed for collection of biofilm biomass which had become suspended into the sterilized groundwater onto a membrane filter with the collection of the filtrate into a separate tube as described for the planktonic microbial community microcosms. The remaining biofilm coupon was transferred to into a scintillation vial. The membrane filters and filtrates were processed as described for the planktonic microbial community samples. Each scintillation vial containing a membrane filter or 1.0 mL filtrate sub-sample received 5.0 mL of Ultima Gold scintillation fluid. The vials containing biofilm coupons received 10.0 mL Ultima Gold. All scintillation vials were set at room temperature in the dark for 6–8 h to stabilize before recording the respective activities on a scintillation counter. The surface area of each biofilm coupon was manually measured after the ^32^P activity had decreased to a safe level.

### Carbon Uptake by Planktonic and Biofilm Microbial Communities

Carbon uptake is the sum of carbon assimilation into biomass and mineralization (i.e., respiration) to CO_2_ and/or CH_4_. Uptake rates can be determined using a mass balance approach with ^14^C-labeled carbon substrates (i.e., acetate) and measuring the ^14^C incorporated into biomass (assimilation), respired CO_2_ and/or CH_4_ and the unincorporated ^14^C-labeled substrate remaining in the sample (Wright and Burnison, 1979).

A volume (200.0 mL) of groundwater was transferred from the pressure vessel as previously described into a sterile and degassed 250 mL polycarbonate flask while under N_2_-gas flow. Sodium acetate (0.831 mM, 68.17 mg L^–1^; 0.333 mM, 4.00 mg L^–1^ as C) and [2-^14^C]-acetate (sodium salt) (23.8 μM, 1.95 mg L^–1^; 9.54 μM, 0.11 mg L^–1^ as carbon) [58.5 mCi mmol^–1^) (ARC Inc., St. Louis, MO, United States)] for an approximate 2.5 × 10^6^ to 3.0 × 10^6^ CPM 10.0 mL^–1^ and gently mixed. Immediately, 10.0 mL sub-samples were transferred to 25 mL sterile and degassed serum bottles (*n* = 18) under continuous N_2_-gas flow, sealed with butyl rubber plugs and aluminum crimp closures. Two sealed bottles were immediately frozen and stored in crushed dry ice (Boyd et al., 2009, 2012; Urschel et al., 2015). The remaining bottles or microcosms were incubated up-side-down at 25–27°C in the dark. At each subsequent time point, two microcosms were frozen as described for the time zero samples. Upon return to the laboratory all frozen samples were stored at −80°C until processed. For the negative controls a replicate set of 10.0 mL dosed samples were added to sparged microcosms containing 500.0 μL of formalin, sealed and incubated as previously described. The negative control samples were processed with the last time point samples.

Frozen samples were slowly thawed at room temperature, then acidified by the injection of 1.0 mL of 1.0 N HCl through each microcosm’s septum. Each acidified sample was connected to a CO_2_ scrubbing system designed to collect ^14^CO_2_ and ^14^CH_4_ produced by the microbial communities, with the ^14^CH_4_ being oxidized to ^14^CO_2_ prior to collection (Nuck and Federle, 1996). Briefly, each acidified microcosm was connected to the gas tight CO_2_ scrubbing system by piercing the microcosm’s plug with a syringe needle connected to a sequence of scintillation vials which are also connected via syringe needles and PTFE tubing as follows: an empty scintillation vial; two scintillation vials containing 5.0 mL of Carbo-Sorb E (PerkinElmer, Waltham, MA, United States) each; a muffle furnace (Lindburg Blue M; Thomas Scientific, Swedesboro, NJ, United States) containing an oxidation process tube filled with cupric oxide pellets and set to 800°C; two scintillation vials containing 5.0 mL of Carbo-Sorb E each. A gas mixture of O_2_/N_2_ (21%:79%) at a flow rate of approximately 40.0 mL min^–1^ for 5 min was used to flush the ^14^CO_2_ and ^14^CH_4_ from the head space of the acidified microcosm through the CO_2_ absorbing solutions.

After flushing each microcosm, the four scintillation vials containing Carbo-Sorb were removed and 6.0 mL of Permafluor E^+^ (PerkinElmer, Waltham, MA, United States) added to each vial and gently mixed. A new set of scintillation vials containing 5.0 mL of Carbon-Sorb E each replaced those removed. The acidified microcosms were removed from the scrubbing system and processed to recover the microbial biomass on membrane filters, retain the filtrate and prepare both for scintillation counting as described for the planktonic microbial communities in the phosphorus uptake experiments.

For the biofilm uptake experiments, groundwater (110.0 mL) from the pressure vessel was filter sterilized as described for the phosphorus uptake by the biofilm microbial community experiments, then dosed with sodium acetate and [2-^14^C]-acetate (sodium salt) to the same final concentration and activity as described planktonic microbial community experiments. Each of nine microcosms (50 mL tubes with septum closures) (Syringa Lab Supplies, Boise, ID, United States) received 10.0 mL of the dosed groundwater. Biofilm growth reactors with the smaller core coupons were recovered, transported, processed and a 2.5 cm long biofilm coupon transferred to each microcosm as described for biofilm microbial community phosphorus uptake experiments. All microcosms were incubated up-side-down at 25–27°C in the dark.

The negative controls were the same as those described for the phosphorus uptake experiments for the biofilm communities, except [2-^14^C] acetate was dosed in place of ^32^PO_4_. Both negative controls were incubated as previously described and processed with the last time point samples.

At each time point, including time zero, one microcosm was immediately frozen and transported in crushed dry ice, then stored at −80°C. The frozen samples were thawed and processed for the recovery of ^14^CO_2_ and oxidized ^14^CH_4_ to ^14^CO_2_ using the scrubbing system, retention of biomass on membrane filters and collection and sub-sampling of filtrates as described for the planktonic microbial community samples. Additionally, the biofilm coupons were transferred to separate scintillation vials to which 10.0 mL Ultima Gold was added. The surface area of each biofilm coupon was manually measured after the final scintillation counts had been performed.

### Nutrient Removal and Uptake Rate Calculations

**FIGURE 2:**
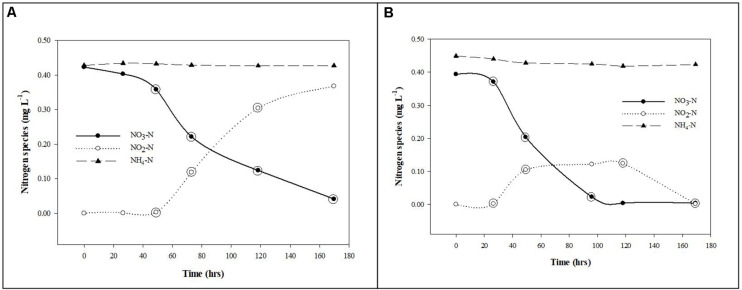
Nitrate removal from groundwater by planktonic and biofilm microbial communities. Trends in NO_3_-N (•), NO_2_-N (∘), and NH_4_-N (▲) removal and production rates by planktonic **(A)** and biofilm **(B)** microbial communities in Upper Floridan Aquifer groundwater. The circled data points were used in the linear regressions to derive the respective removal or production rates.

The nitrogen species removal and production rates were calculated from the slopes of the regression lines using the linear segments of the plotted data for the planktonic and biofilm communities (Figure 2).

**FIGURE 3:**
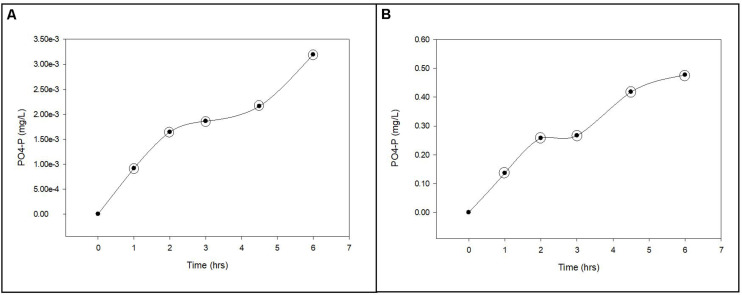
Phosphorus uptake from groundwater by planktonic and biofilm microbial communities. Trends in PO_4_-P uptake by planktonic **(A)** and biofilm **(B)** microbial communities in Upper Floridan Aquifer groundwater. The circled data points were used in the linear regressions to derive the respective uptake rates.

**FIGURE 4:**
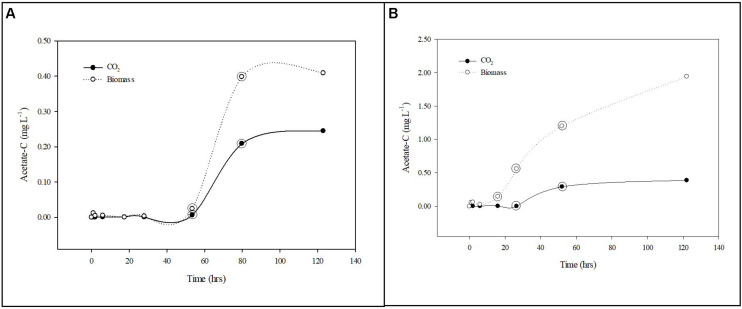
Carbon uptake from groundwater by planktonic and biofilm communities. Trends in acetate-C mineralization to CO_2_ (•) and assimilation into biomass (∘) by planktonic **(A)** and biofilm **(B)** microbial communities in Upper Floridan Aquifer groundwater. The circled data points were used in the linear regressions to derive the respective uptake rates.

Rates of phosphorus and carbon uptake (*v*) by planktonic and biofilm bacterial communities were calculated using the following equation (Wright, 1974; Wright and Burnison, 1979):

(1) $$v=\frac{f\,\left(S_{\text{n}}+A\right)}{t}$$

where *f* is the decimal fraction of the activity incorporated into biomass (assimilation) and CO_2_ (respiration) at each time point relative to total activity added to the solution at time zero using scintillation counts of the biomass (planktonic and biofilm), CO_2_ (when applicable) and filtrate samples; *S*_n_ is the background or dosed non-radiolabeled nutrient concentration; *A* is the dosed radiolabeled nutrient concentration; *t* is incubation time. All scintillation counts used in the calculations were normalized by subtracting the appropriate control sample scintillation counts before conversion to concentrations. The normalized scintillation counts were converted to concentrations and the individual uptake rates (*v*) were calculated for each time point within the linear segments of the uptake curves for phosphorus and carbon. The individual uptake rates were then used to calculate the respective mean (±standard deviation) uptake rates. The general trends in ^32^P and ^14^C uptake by suspended and biofilm associated cells are presented in Figures 3, 4.

### Total Cell Counts

Separate 50.0 mL samples were collected directly from the well head in parallel with the larger volumes collected for the respective nutrient uptake experiments. These samples were immediately preserved by adding 0.5 mL of filter sterilized formalin and stored at 4°C until processing. Equal volumes of each preserved sample were filtered through replicate 25 mm diameter, 0.2 μm pore size filters (GTPB, Millipore Corp.) to retain the bacterial biomass. The bacteria were labeled using SYBR Gold (supplied at 10,000×, final concentration 25×) (Invitrogen) as described by Lisle and Priscu (2004). Labeled bacteria were counted on an Olympus BX51 epifluorescent microscope, equipped with a filter cube optimized for SYBR Gold (λ_Ex_ = 480 nm; λ_E__m_ = 535 nm; λ_B__s_ = 505 nm), at a final magnification of 1,250×. A minimum of 300 bacterial cells were counted in a minimum of 20 microscope fields per filter. Due to significant amounts of carbonate core material being associated with the biofilm samples during the removal procedure it was not possible to proficiently separate the cells from the carbonate mud to the point where reliable cell counts could be determined.

## Results

### Native Groundwater Chemistry and Bacterial Abundance

The zone of the UFA accessed is anaerobic and significantly reduced (−289.15 – −227.65 mV), with moderate temperature (25.29–25.83°C) and pH (7.68–8.10). Additionally, this zone can be classified as oligotrophic as NO_2_-N, NO_3_-N, and PO_4_-P are below the methodological detection limits and other terminal electron acceptors (i.e., Mn^2+^ and Fe^2+^) and dissolved organic carbon (1.12–1.68 mg L^–1^) are present at relatively low concentrations (Table 1). This zone of the UFA is colonized with a planktonic bacterial community at an abundance of 1.40 × 10^4^ ± 1.15 × 10^4^ cells mL^–1^ (2.48 × 10^3^–2.54 × 10^4^ cells mL^–1^). Biofilm cell abundance counts were not performed.

### NO_3_-N Removal Rates

After an approximate 20 h acclimation period, the planktonic microbial community removed NO_3_-N at a rate of 0.059 mg L^–1^ d^–1^ (*p*-value: 0.036; *r*^2^: 0.893), with a concomitant NO_2_-N production rate of 0.103 mg L^–1^ d^–1^ (*p*-value: 0.026; *r*^2^: 0.997) (Table 2 and Figure 2A). The NH_4_-N concentrations during this same time interval did not significantly change (0.430 ± 0.003 mg L^–1^) (Figure 2A).

**TABLE 2:** Experimental nutrient removal and uptake rates for Upper Floridan Aquifer planktonic and biofilm microbial communities.

**Microcosm**	**Analytical target**	**Planktonic removal and uptake rates**	**Biofilm removal and uptake rates**		
		**(mg⋅L^–1^) d^–1^**	**(mg⋅L^–1^) d^–1^**	**(m^2^)^1^**	**(mg⋅L^–1^) m^–2^ d^–1^**
NO_3_-N	Net removal	0.059	0.116	12.85–72.37	0.0016–0.0090
PO_4_-P	Biomass	5.73 × 10^–8^–1.03 × 10^–7^	4.20 × 10^–5^–5.91 × 10^–5^	4.25–23.92	2.47 × 10^–6^–9.88 × 10^–6^
DOC (as acetate)	CO_2_	0.003–0.084	0.001–0.014	4.25–23.92	3.29 × 10^–4^–5.77 × 10^–4^
	Biomass	0.012–0.160	0.299–0.682	4.25–23.92	0.028–0.070
	Total	0.015–0.244	0.301–0.696	4.25–23.92	0.029–0.071

*^1^The biofilm coupon surface areas were calculated using specific gravity (2.70–2.83 g cm^−3^) and surface area:mass ratio (0.8–4.3 m^2^ g^–1^) values.*

The biofilm microbial community also initiated NO_3_-N removal after approximately 20 h at a rate of 0.116 (mg L^–1^) d^–1^ (*p*-value: 0.111; *r*^2^: 0.940) (Table 2), which was approximately twofold greater than the planktonic microbial community, to below detection limit concentrations after approximately 120 h (Figure 2B). During this same period, NO_2_-N was generated at a rate [0.108 (mg L^–1^) d^–1^] similar to that for the planktonic microbial community. However, and in contrast to the planktonic microbial community, an approximate 3 days period of no net change in the NO_2_-N concentration was followed by a NO_2_-N removal phase [0.057 (mg L^–1^) d^–1^] to concentrations below detection limits (Figure 2B).

Normalizing the removal rates to surface area estimates for the core coupons, the NO_3_-N removal rates ranged from 0.0016–0.0090 (mg L^–1^) m^–2^ d^–1^ (Table 2). The initial NO_2_-N production rate of 0.0015–0.0084 (mg L^–1^) m^–2^ d^–1^ was similar to the NO_3_-N removal rate over the same time interval (Figure 2B). However, and in contrast to the planktonic microbial community data, an approximate 3.0 days period of no net change in NO_2_-N concentration was followed by removal at a rate of 0.0008–0.0044 (mg L^–1^) m^–2^ d^–1^ (Figure 2B). As in the planktonic microbial community microcosm, there was no significant change in the NH_4_-N concentrations (0.431 ± 0.011 mg L^–1^) over the duration of the experiments (Figure 2B).

### PO_4_-P Uptake and Sorption Rates

The uptake rates of PO_4_-P into the planktonic microbial community biomass was between 5.73 × 10^–8^ and 1.03 × 10^–7^ mg L^–1^ d^–1^ (Table 2 and Figure 3A). The biofilm microbial community incorporated PO_4_-P into biomass at a rate approximately 2.8-orders of magnitude higher (4.20 × 10^–5^–5.91 × 10^–5^ mg L^–1^ d^–1^) than the planktonic microbial community (Table 2 and Figure 3B). Normalizing these uptake rates to the estimated surface areas within the core coupons, the uptake rates ranged from 2.47 × 10^–6^–9.88 × 10^–6^ (mg L^–1^) m^–2^ d^–1^ (Table 2).

The PO_4_-P sorption rates onto the carbonate coupons can be estimated ^32^P activity associated with the formalinized negative controls (So et al., 2011). Using the same calculations as used for the uptake rates, where *v* would now represent sorption rates, the derived PO_4_-P sorption rate ranged from 1.64 × 10^–7^ to 9.25 × 10^–7^ mg PO_4_-P m^–2^ day^–1^. These sorption rates are 10.7–15.1-fold lower than the biofilm community uptake rates.

### Carbon Uptake Rates

After a transition period of 53.5 h, during which there was no detectable uptake of ^14^C-labeled acetate, the planktonic microbial community respired and assimilated the acetate-C at rates of 0.003–0.084 mg L^–1^ d^–1^ and 0.012–0.160 mg L^–1^ d^–1^, respectively, for an uptake rate of 0.015–0.244 mg L^–1^ d^–1^ (Table 2 and Figure 4A).

The biofilm microbial community respiration response was detectable after 28.0 h [0.001–0.014 (mg L^–1^) d^–1^] and was between two and sixfold lower than that for the planktonic microbial community (Table 2 and Figure 4B). The assimilation response initiated at 16.0 h [0.299–0.682 (mg L^–1^) d^–1^] and, in contrast to the respiration rates, was 4- to 25-fold greater than that for the planktonic microbial community (Table 2). Collectively, the biofilm microbial community respiration and assimilation rates provide an acetate-C uptake rate of 0.301–0.696 (mg L^–1^) d^–1^, which is 3- to 20-fold greater than the uptake rates for the planktonic microbial community (Table 2). Normalizing these uptake rates to the estimated surface areas of the core coupons the rates of respiration [3.29 × 10^–4^–5.77 × 10^–4^ (mg L^–1^) m^–2^ d^–1^] and assimilation [0.028–0.070 (mg L^–1^) m^–2^ d^–1^] the biofilm microbial community uptake rate was 0.029–0.071 (mg L^–1^) m^–2^ d^–1^ (Table 2).

Additionally, both microbial communities incorporated a greater percentage of carbon into bacterial biomass when compared to produced CO_2_, indicating a preference for productivity under these conditions. The planktonic community incorporated carbon into biomass at a rate of 1.9–4.0-fold greater than that for CO_2_ production, while the biofilm community rate was 85.1–121.3-fold greater (Table 2).

There was no ^14^C-labeled CH_4_ detected in any sample, using the CH_4_ oxidation to CO_2_ method described previously.

### Comparison of Experimental and ASR Cycle Test Nutrient Removal and Uptake Rates

Applying the nutrient removal or uptake rates derived from this study (Table 2) and the estimated biofilm surface area within the UFA storage zone, the time required for the planktonic and biofilm communities to remove nutrients during the storage phase in the UFA at KRASR can be estimated, assuming no mixing with native groundwater occurs during this period (Mirecki et al., 2013). For example, using averaged data from four cycle tests at the KRASR facility (Mirecki, 2013) and applying the range of porosities (0.25–0.30), carbonate specific gravity (2.70–2.83 g cm^–3^) and surface area:mass ratio (0.8–4.3 m^2^ g^–1^) values, as previously described, the following variables describe an average ASR cycle: (a) the storage zone is 69.5 m deep × 186.2–204.2 m radial distance from the injection well; (b) giving a storage zone volume of 7.57 × 10^6^–9.10 × 10^6^ m^3^ or a mass of 2.04 × 10^13^–2.57 × 10^13^ g; (c) the total surface area within this storage zone volume available for biofilm colonization is 1.64 × 10^13^–1.11 × 10^14^ m^2^; (d) only 10% of the total surface area is colonized with biofilms (1.64 × 10^12^–1.11 × 10^13^ m^2^); (e) the recharge water volume is 2.27 × 10^9^ L (6.00 × 10^8^ gallons); (f) storage period of 150 days. The average nutrient concentrations in the recharge and recovered water and removal rates, based on 150 days of storage, are listed in Table 3.

**TABLE 3:** Average nutrient concentrations and removal rates from cycle test data at Kissimmee River ASR facility^1^.

**Nutrient**	**Recharge water**	**Recovered water**	**Removed during storage**	**Removal rate during storage**
	**(mg⋅L^–1^)**	**(mg⋅L^–1^)**	**(mg⋅L^–1^)**	**(mg⋅L^–1^) d^–1^**
NO_3_-N	0.47	0.00	0.47	3.13 × 10^–3^
PO_4_-P	0.059	0.011	0.048	3.19 × 10^–4^
DOC	18.0	1.6	16.4	0.11

*^1^Assuming a storage period of 150 days.*

The planktonic microbial community removal and uptake rates (Table 2) are directly comparable to those calculated from the KRASR cycle test data (Table 3), when both sets of rates are normalized to a 150 days storage period. The experimental NO_3_-N removal rate was approximately 20-fold greater than the rate from the averaged cycle test data, taking 8.0 days within the 150 days storage period to remove the same concentration of NO_3_-N (Table 4). However, the experimental PO_4_-P uptake rates (Table 2) were significantly lower than the rates from the cycle tests, taking years to remove the same concentration of PO_4_-P as recorded during the cycle tests (Table 4). The range of total DOC (i.e., CO_2_ + biomass) uptake rates ranged from approximately 7.3-fold lower to 2.2-fold greater than the average cycle test rate, thereby requiring between approximately half of the 150 days storage period (73 days) and 3.0 years to remove the same concentration of DOC (Table 4).

**TABLE 4:** Time or biofilm area required to remove the same concentrations of nutrients in recharged surface water as removed during an average cycle test at the Kissimmee River ASR facility.

**Nutrient**	**Planktonic community**	**Biofilm community**	
	**Days^a^ or years^b^**	**m^2^**	**% of biofilm area**
NO_3_-N	8.0^a^	7.93 × 10^8^–4.46 × 10^9^	0.04–0.05
PO_4_-P	1.28 × 10^3^–2.30 × 10^3^ ^b^	5.99 × 10^10^–2.39 × 10^11^	2.15–3.65
DOC (total)	0.2–3. ^b^	3.18 × 10^9^–7.80 × 10^9^	0.07–0.19

The comparison between the average nutrient removal rates from the KRASR cycle tests and the respective biofilm microbial community rates is not direct as with the planktonic microbial community rates. For this example, deriving the percentage of biofilm colonized surface area needed to remove or uptake the same concentrations of nutrients as removed during the cycle test storage period of 150 days is the objective. To facilitate these comparisons, the concentrations of nutrients removed during cycle test storage (Table 3) were converted to mass in the storage zone for NO_3_-N (1.07 × 10^9^ mg), PO_4_-P (8.87 × 10^7^ mg), and total DOC (3.39 × 10^10^ mg). Thereby, applying the nutrient removal and uptake rates for the biofilm microbial community (Table 2), the area of the total biofilm colonized aquifer surfaces required to remove the same mass of nutrients as during the complete 150 days storage period of the cycle test would be <1.0% for NO_3_-N, 2.15–3.65% for PO_4_-P and <1.0% for total DOC of the 1.64 × 10^12^–1.11 × 10^13^ m^2^ of biofilm in the storage zone (Table 4).

## Discussion

To date, estimates of nutrient reductions during storage have relied on data collected at the surface from the recharge water prior to injection and after a storage period. This “black box” approach necessitates the application of indirect estimates and hypotheses to explain the geochemical and microbial processes responsible for these changes at depth. Where other studies have considered the microbial contribution to these nutrient removal rates as being a single, collective community within the aquifer storage zone, this study partitions this community into two ecological niches: planktonic and biofilm.

In general, the biofilm communities removed all nutrients at significantly greater rates than the planktonic communities (Table 2). These greater rates of nutrient removal by the biofilm communities is assumed to be the result of the greater microbial biomass associated with this niche, relative to the planktonic communities. Though not determined during this study, biofilms have also been shown to support microbial groups with physiological capabilities for the metabolism of nutrients that are not associated with the planktonic communities (Nadell et al., 2016; Stubbendieck et al., 2016; Jones and Bennett, 2017).

When comparing the removal rates between these two niches based on a nutrient removal rate per day basis, the biofilm communities removed NO_3_-N, PO_4_-P and DOC (as acetate) at rates approximately 2-fold, 570 to 733-fold and 3 to 20-fold greater than the planktonic communities, respectively. However, nitrogen, phosphorus and carbon do not cycle independently of other elements as most biogeochemical processes, especially carbon cycling, are intra- and interconnected (Taylor and Townsend, 2010; Anderson, 2018; Hofmann and Griebler, 2018).

Due to this zone of the UFA being anaerobic, NO_3_-N removal in the planktonic and biofilm communities is assumed to be the result of denitrification and dissimilatory nitrate/nitrite reduction to ammonium (DNRA), both heterotrophic processes, and to a lesser degree autotrophic denitrification (Lam and Kuypers, 2010; Kuypers et al., 2018). Using the native DOC in the UFA (∼1.40 mg/L) (Table 1), the planktonic communities removed NO_3_-N with a concomitant production of NO_2_-N at a stoichiometric ratio of approximately 0.6:1 during the mid-to-late time points of the study (Figure 2A). The relatively consistent accumulation of NO_2_-N without the production of NH_4_-N suggests nitrate reduction, the first step in denitrification, was the dominant process while bacterial groups responsible for nitrite reduction to either NO, N_2_ (i.e., complete denitrification), NH_4_-N (i.e., dissimilatory nitrate/nitrite reduction to ammonium; DNRA) or oxidation of NH_4_-N (i.e., anaerobic ammonium oxidation; anammox) were either not present or at abundances too low to remove NO_2_-N and produce NH_4_-N at rates reliably measured by the methods used in this study (Lam and Kuypers, 2010; Taylor and Townsend, 2010; Kuypers et al., 2018).

The biofilm community reduced NO_3_-N to NO_2_-N at a rate approximately twofold greater than the planktonic community during the initial phase of the study (Figure 2B). The plateau in the NO_2_-N production is accompanied with a slight decrease in NH_4_-N, suggesting annamox is present but denitrification is dominant until NO_3_-N is completely removed and denitrification removes the remaining NO_2_-N. This opposing trend, relative to the planktonic community, suggests the biofilm communities included cells or groups of cells at abundances high enough and spatially positioned within the biofilms to complete the denitrification process and possibly anammox (Elias and Banin, 2012; Liu et al., 2016). The likelihood of denitrification being the dominant and anammox the relatively minor contributors to the nitrogen cycle in the planktonic and biofilm microcosms is increased by the presence of sulfides in the UFA groundwater (Table 1) at concentrations that have been shown to suppress rates of anammox while having no effect on denitrification rates (Carvajal-Arroyo et al., 2013).

Preliminary experiments had shown negligible PO_4_-P uptake by planktonic communities when using unamended native UFA groundwater, though it contained adequate concentrations of TOC and DOC (Table 1). It was only after the addition of acetate that the planktonic and biofilm communities actively incorporated phosphorus into biomass (Figures 3A,B), indicating the native TOC and DOC in the UFA (Table 1) is recalcitrant and not readily accessible for facilitating the microbial uptake of phosphorus. The coupling of bacterial carbon and phosphorus cycles in aquatic ecosystems has been shown to be an important biogeochemical relationship that imposes partial controls on bacterial productivity (Dorado-Garcia et al., 2014; Anderson, 2018; Hofmann and Griebler, 2018). The cooperative relationship between bacterial access to carbon and phosphorus uptake is the positive relationship between the initiation or increase in free and cell-bound alkaline phosphatase activity and increasing labile carbon concentrations (Anderson, 2018). This increase in alkaline phosphatase activity increases the rate at which phosphate groups are cleaved from complex organic and inorganic compounds outside the bacterial cell or between the cell wall and periplasmic membrane with the subsequent transport of the inorganic phosphate group into the bacterial cell for assimilation (Jansson, 1988). The significantly greater PO_4_-P uptake rates by the biofilm communities, relative to the planktonic communities, can be attributed to a greater abundance of bacterial cells actively producing free and cell-bound alkaline phosphatase and the ability of biofilms to retain and concentrate cellular metabolites (Jefferson, 2004; Elias and Banin, 2012). Bacterial biofilms have been shown to not only retain alkaline phosphatase at relatively higher concentrations than the overlying water but also promote higher enzyme activity within the biofilm matrix (Huang et al., 1998).

The rates of PO_4_-P sorption onto and desorption from the aquifer core coupon are significant factors when assessing the capacity of an aquifer storage zone to retain this nutrient. A previous study that used carbonate core material, similar to that used in this study, from a surficial aquifer in south Florida derived a sorption rate for PO_4_-P in seawater (Price et al., 2010). After normalizing their sorption rates for direct comparison to those in this study by using the density and surface area:mass ratios described previously, their PO_4_-P sorption rates of 1.79 × 10^–7^–4.54 × 10^–6^ mg PO_4_-P m^–2^ day^–1^ were similar to those derived in this study (1.64 × 10^–7^–9.25 × 10^–7^ mg PO_4_-P m^–2^ day^–1^).

With respect to diversity, it is worthy of note that the nutrient removal and uptake rates described in this study are community-level rate estimates for bacterial populations that have not been impacted by injected treated or untreated surface water. An understanding of changes in the proficiency of nutrient removal during storage of recharged water by planktonic and biofilm communities will require a more detailed characterization of those communities to identify those populations actively cycling those nutrients and the succession of bacterial diversity and function.

In addition to monitoring changes in constituents, including microbial communities, in the recharge and recovered water at an ASR, or any MAR facility, the characterization of biofilms within the aquifer storage zone prior to and during recharge and recovery cycles need to be included if the fate and transport of nutrients and the impact on operational metrics (e.g., well clogging) are to be adequately modeled. However, the application of microbial diversities and rates of biogeochemical processes generated at one ASR location to another location and/or different MAR technology should be attempted with caution if the geochemical (e.g., oxidized, reduced, anoxic, and anaerobic), mineralogical (e.g., presence or absence of iron) and hydrological (e.g., rates of mixing between recharged water and native groundwater within the storage zone) conditions are too dissimilar.

## Data Availability Statement

The datasets generated for this study are available on the USGS Data Release portal (doi: 10.5066/P9EOM5RC).

## Author Contributions

JL conceived and designed the experiments, performed the samplings, analyzed the data, and wrote the manuscript.

## Conflict of Interest

The authors declare that the research was conducted in the absence of any commercial or financial relationships that could be construed as a potential conflict of interest.

## Footnotes

1 https://automeris.io/WebPlotDigitizer

2 https://floridadep.gov/fgs/geologic-collections/content/core-and-cuttings-repository
